# Origin of Structural Transformation in Mono- and Bi-Layered Molybdenum Disulfide

**DOI:** 10.1038/srep26666

**Published:** 2016-05-26

**Authors:** Xiaoli Sun, Zhiguo Wang, Zhijie Li, Y. Q. Fu

**Affiliations:** 1 School of Physical Electronics, University of Electronic Science and Technology of China, Chengdu, 610054, P.R. China; 2 Department of Physics and Electrical Engineering, Faculty of Engineering and Environment, University of Northumbria, Newcastle upon Tyne, NE1 8ST, UK

## Abstract

Mono- and multi-layered molybdenum disulfide (MoS_2_) is considered to be one of the next generation anode materials for rechargeable ion batteries. Structural transformation from trigonal prismatic (2H) to octahedral (1T) upon lithium or sodium intercalation has been *in-situ* observed experimentally using transmission electron microscope during studies of their electrochemical dynamics processes. In this work, we explored the fundamental mechanisms of this structural transformation in both mono- and bi-layered MoS_2_ using density functional theory. For the intercalated MoS_2_, the Li and Na donate their electrons to the MoS_2_. Based on the theoretical analysis, we confirmed that, for the first time, electron transfer is dominant in initiating this structural transformation, and the results provide an in-depth understanding of the transformation mechanism induced by the electron doping. The critical values of electron concentrations for this structural transformation are decreased with increasing the layer thickness.

Currently, graphite is the main anode materials for commercial lithium ion batteries (LIBs) due to its ability to cause reversible intercalation/deintercalation of Li^+^ ions in the layered structure^1^. However, its low Li storage capacity (372 mAhg^−1^) cannot satisfy the large power demanding for electric vehicles and smart grids^2^,^3^. Transition metal dichalcogenides (TMDs) with graphite-like layered structures, such as WS_2_^4^, MoS_2_^5^,^6^, MoSe_2_^7^, TiS_2_^8^ have received tremendous attention as alternatives to graphite for the anode materials in the rechargeable ion batteries. In the layered TMDs, anions constitute hexagonal close-packed layers, and transition metals are sandwiched between layers of anions to form two-dimensional layers with atoms covalently bonded. The two-dimensional layers are stacked together through weak van der Waals interactions between the TMD layers^9^, which allows the Li^+^ and Na^+^ ions to diffuse without a significant increase in volume expansion and thus prevents the pulverization problem of active materials caused by the repeated intercalation/deintercalation. The layered TMDs such as MoS_2_ have attractive specific capacities of Li storage, for example, MoS_2_/graphene nanocomposites exhibited a high specific capacity of 1225–1400 mAh/g^10^,^11^, and still had a capacity of 1351 mAh/g after 200 repeated charge-discharge cycles^12^.

The TMDs have a variety of polytypic structures depending on the arrangement of the chalcogen atoms. The transition metal atoms have six-fold coordinates and are hexagonally packed between two trigonal atomic layers of chalcogen atoms. One polytype is based on trigonal symmetry (2H), where the chalcogen atoms are located in the lattice positions of a hexagonal close-packed structure. The metal atoms are sandwiched between two atomic layers of chalcogen in a trigonal prismatic geometry. Another polytype is based on the metal atoms octahedrically or disordered octahedrically located in the environment of the chalcogen atoms. As shown in Fig. 1, the layers are composed by prismatic D_3h_-, octahedral O_h_-, and octahedral O_h_-disordered MoS_2_ units, which are termed as 2H-, 1T- and 1T’-MoS_2_, respectively^13^. The electronic properties of the TMDs show a significant dependence on the polytypic structures^13^, for example, the 2H-MoS_2_ phase shows a semiconductor nature, whereas the 1T-MoS_2_ phases show a metallic character^6^,^14^,^15^. The electronic properties of the TMDs can be tuned by applying strain^16^ or formation of heterostructures^17^,^18^.

MoS_2_ and its associated composites have been investigated as anode materials for rechargeable LIBs and sodium ion batteries^19^,^20^,^21^,^22^,^23^ through intercalation mechanisms. As mentioned above, 2H-MoS_2_ has a stable crystal structure with a semiconductor character^24^, whereas the metastable 1T/1T’-MoS_2_ phase was introduced inside the 2H-MoS_2_ by intercalating alkali metals^25^. Using *in-situ* transmission electron microscopy (TEM) technique, a real time imaging characterization of the electrochemical process at the atomic level was performed to investigate the atomistic mechanisms of the 2H-1T/1T’ transition in the MoS_2_ upon lithium or sodium intercalation^26^,^27^,^28^. A shear mechanism of the 2H-1T/1T’ phase transition has been identified by probing the dynamic phase boundary movement^27^. The stability of the 2H- and 1T-LiMoS_2_ has also been investigated as functions of the Li content and intercalation sites^29^,^30^, and results showed that the critical content of lithium, required for the initialization of the 2H→1T phase transition, was estimated to be x ≈ 0.4 in Li_x_MoS_2_^29^.

Apart from the alkali metals, whose intercalation could induce 2H→1T/1T’ phase transition, the phase transition in the MoS_2_ was also reported to be caused by the substitutional doping of Mo by Re atom^31^, in which Re has one more valence electron than Mo. The 2H-1T’ phase transition was also reported to be induced by using a high dose electron beam irradiation during heating the MoS_2_ monolayer^32^ or by using hot electrons generated by plasmonic nanoparticles deposited onto a MoS_2_ monolayer^33^.

However, currently the mechanisms of the structural transformation from 2H→1T/1T’ induced by various methods, such as alkali metals intercalation, Re-doping, electron irradiation and hot electron doping, are not fully understood. As the metastable 1T-MoS_2_ shows enhanced magnetism^34^ and can be used as electrode materials for supercapacitors^35^, understanding the mechanisms of these structural transformations is crucial to improve the battery performance, material design and practical applications.

The MoS_2_ shows layer-dependence electronic properties^36^,^37^,^38^. The valence bands of the monolayer MoS_2_ are distinctly different from those of few-layer and bulk MoS_2_, and the valence band maximum of a MoS_2_ monolayer is located at *K* point of the first Brillouin zone (BZ), rather than at *Г* point in a bulk MoS_2_^36^. Electrocatalysis of the MoS_2_ for hydrogen evolution also showed this layer dependent behaviour^39^. If the layered MoS_2_ is used in the anode materials for rechargeable ion batteries, the interstitial sites between the adjacent layers provide different adsorption sites compared with those of a monolayer MoS_2_. The MoS_2_ materials studied in the literature have various properties of size, morphology and number of layers^19^,^20^,^21^,^22^,^23^. The dependence of structural transformation on the layer number has not been investigated. Therefore, it is imperative to obtain a comprehensive understanding of the structural transformation in different layered MoS_2_.

In this paper, for the first time, the origin or mechanism of the structural transformation of mono-and bi-layers MoS_2_ was investigated using a density functional theory (DFT). Based on the results from the first principle calculation, we concluded that the electron transfer is the key reason for the structural transformation of the 2H→1T’ in the MoS_2_.

## Results

The lattice parameters of the 2H-MoS_2_ mono-and bi-layers after a full structural optimization using the DFT are *a* = *b* = 3.19 Å, which are consistent with the previously calculated values of 3.18–3.19 Å^40^,^41^ and experimental value of 3.20 Å^42^. Those of the 1T’-MoS_2_ are *a* = *b* = 3.18 Å. It was reported that there are several types of stacking sequences for the bilayer MoS_2_ synthesized using chemical vapour deposition method^43^,^44^,^45^. Changing the stacking sequence can tune the electronic properties of the bilayer MoS_2_. The DFT simulations showed that the bilayer MoS_2_ with AA’ stacking sequence is energy favorable than the other types of stacking sequences^46^. In AA’ stacking sequence, the top layer Mo (S) atoms align vertically with the bottom layer S (Mo) atoms. In this work, we modeled the structural transformation of the bilayer MoS_2_ with AA’ stacking sequence.

### 2H→1T’ phase transition in MoS_2_ upon electron doping

**Figure 1:**
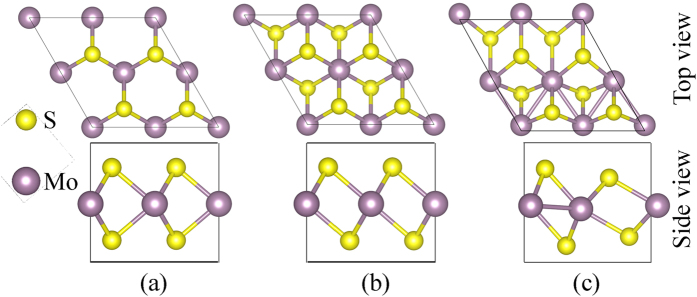
Atomistic configuration of MoS_2_. Top and side views of the (**a**) 2H-, (**b**) 1T- and (**c**) 1T’-MoS_2_. The Mo atoms have octahedral and trigonal prismatic coordination in the 1T/1T’- and 2H-MoS_2_, respectively.

**Figure 2:**
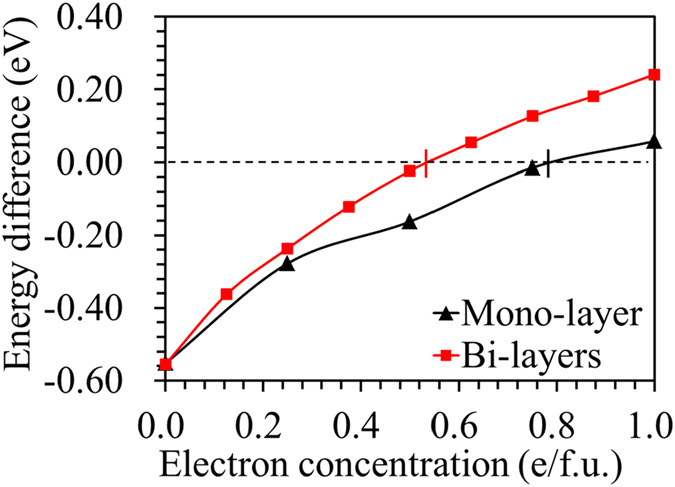
Energy stability of 2H- and 1T’-MoS_2_. Energy difference per MoS_2_ molecular between the 2H- and 1T’-phase as a function of extra electron concentration. The critical extra electron concentrations for the 2H→1T’ phase transition are 0.55 and 0.78 e/f.u. in mono- and bi-layers, respectively.

A 2 × 2 hexagonal supercell of the MoS_2_ layers was used to study the stability of both the 2H- and 1T’-MoS_2_. The 1T-MoS_2_ monolayer can maintain its structure with a 1 × 1 supercell, however, it will change into the 1T’ structure when a 2 × 2 supercells was used. This phenomenon was also reported by Kan *et al.*^47^. First principles analysis shows that the instability of the 1T-MoS_2_ is caused by the instability of phonon dispersion at M-point^48^. A distorted structure of 1T-MoS_2_ phase, i.e. the 1T’-MoS_2_, can be stabilized by dimerization of Mo atoms^48^,^49^,^50^, as shown in Fig. 1(c). The calculated three nearest Mo-Mo distances are 2.775, 3.193, and 3.825 Å, which agree with the previous simulation values of 2.769, 3.175 and 3.808 Å^51^. Based on the analysis, we did not find any layer dependent dimerization of the Mo atoms. The 1T’-MoS_2_ is 0.26 eV per formula unit (eV/f.u.) energy lower than that of the 1T-MoS_2_ for both mono- and bi-layers. To investigate the stability of both the 2H- and 1T’-LiMoS_2_, extra numbers of electrons were injected into the MoS_2_ lattices instead of the traditional method of increasing the Li adsorption to characterize the modified electron density^29^,^30^. Figure 2 shows the energy difference per MoS_2_ molecule between the 2H- and 1T’-phases, Δ*E* = *E*_1T’_ − *E*_2H_, as a function of extra electron concentration. The 2H-phase is more stable than 1T’-phase at lower electron concentrations, and it is also energetically stable (with an energy difference value of 0.54 eV/f.u.) than the 1T’-phase without addition of electrons, which agrees well with the value of 0.55 eV/f.u. reported by Esfahani *et al.*^30^ and 0.51 eV/f.u. reported by Kan *et al.*^47^. The 1T’-phase becomes more stable with increasing the electron concentration, i.e. a 2H→1T’ phase transition will occur by increasing the electron concentration. The critical values of adding extra electron concentrations to trigger the 2H→1T’ phase transition were calculated to be 0.78 and 0.55 e/f.u. for the mono- and bi-layers, respectively. For the bulk Li_*x*_MoS_2_, the critical value of *x* was predicted to be 0.4 for the 2H→1T structural transformation^29^. Therefore, our results showed that the critical electron concentration for the 2H→1T’ phase transition decreases with the increase of thickness of MoS_2_ layers.

### Adsorption of Li/Na on 2H-MoS_2_

**Figure 3:**
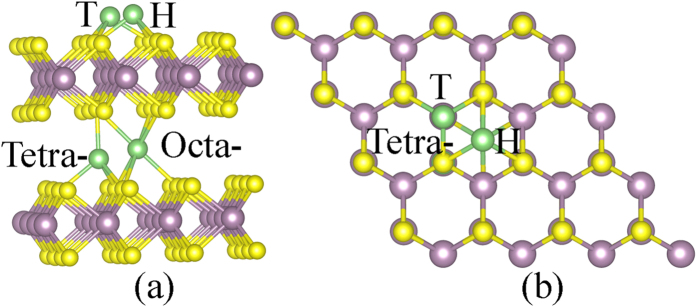
Atomistic configuration of Li/Na adsorbed MoS_2_. (**a**) Side-view and (**b**) cross-section views of the possible adsorption sites for Li/Na in bi-layers 2H-MoS_2_.

Li/Na adsorptions on the mono- and bi-layers 2H-MoS_2_ were investigated using a 6 × 6 MoS_2_ hexagonal supercell to avoid periodical image interactions. All the previous investigations^41^,^42^ showed that both the Li and Na prefer to occupy the top of the molybdenum site (T) compared with center of the hexagon site (H) on the mono-layer of the 2H-MoS_2_. There are two preferred positions for the Li/Na intercalation into the interlayer spaces for MoS_2_ bi-layers: (1) an octahedral site enclosed by six S atoms; and (2) a tetrahedral site enclosed by four S atoms. These interstitial sites are corresponding to the T and H sites in the monolayer MoS_2_. Figure 3 shows the side-view and cross-section view of the adsorption sites. We calculated the adsorption energy values of Li/Na on the MoS_2_ using *E*_ads_ = *E*_MoS2+Li/Na_ − *E*_MoS2_ − *E*_Li/Na_, where *E*_MoS2+Li/Na_ and *E*_MoS2_ are the total energies of MoS_2_ with and without Li/Na adatom adsorption, respectively. The adsorption energy can be calculated reference to adatom either in vacuum (modeled as an isolated atom in a supercell of size 30 × 30 × 30 Å^3^) or in bulk metal. *E*_Li/Na_ is the energy of an isolated Li/Na atom or half of the energy body center cubic Li/Na bulk metal. A negative value of the adsorption energy indicates a thermodynamic favorable intercalation process.

The calculated adsorption energies of the Li/Na in the monolayer and bilayer 2H-MoS_2_ are listed in Table 1. The calculated adsorption energies are −1.8 and −1.6 eV for the Li to be adsorbed at T and H sites on mono-layer 2H-MoS_2_, respectively, which agree well with the previous report of Li prefer to occupy the T site^52^,^53^. The adsorption energy of the Na adsorbed at the T site on the 2H-MoS_2_ is −1.3 eV, which is 0.1 eV energy lower than that adsorbed at the H site. It was reported that the Na cannot penetrate through the surface monolayer of MoS_2_, and it prefers to stay on the surface of (0001) of MoS_2_^54^. whereas K can be intercalated into the interlayer spaces of MoS_2_ crystal^55^.

It was found that the adsorption energy value of the octahedral site is 0.12 eV lower than that of the tetrahedral site for Na adsorbed in the bi-layers of the 2H-MoS_2_. However, the Li prefers to occupy the tetrahedral site. It was also obtained that the Li and Na all prefer to occupy the interlayer position than the surface of the 2H-MoS_2_. Previous simulation results also showed that the Li prefers to be in the interlayer space than on the surface in bi-layers graphene^56^.

### Charge distribution in MoS_2_ upon electron doping and Li/Na adsorption

The effects of extra numbers of electrons by the electron injection were studied using the equation (1) based on the differences in charge densities in the MoS_2_ with and without electron doping,


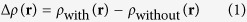


where 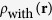 and 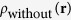 are the charge densities of the MoS_2_ with and without electron injection at position ***r***, respectively. The electron injection was performed by adding electrons into the cell, and a compensating background was used to achieve the charge neutrality^57^. This was done by immerging the original charged system into a jellium background which fills the cell, and then neutralizing the charge to keep the net charge to be zero^58^. The redistribution of charge densities of Li/Na adsorbed MoS_2_ systems was calculated using the equation (2),


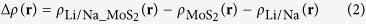


where 
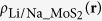
 and 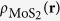 are the space charge densities of the MoS_2_ with and without Li/Na adsorption, respectively. 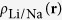 is the electron charge density of an isolated Li/Na at the same position in the supercell as in the Li/Na- MoS_2_ systems.

**Figure 4:**
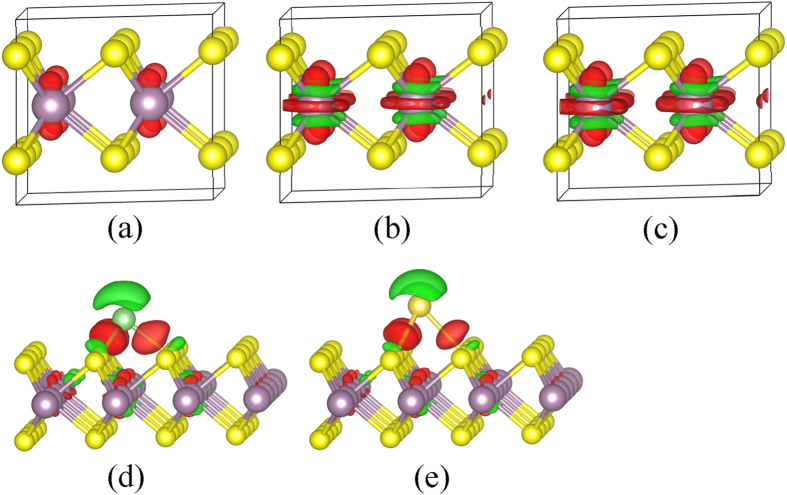
Charge distributions of monolayer 2H-MoS_2_. Isosurface (0.003 e/Å^3^) of the charge distributions of 2H-MoS_2_ doped with (**a**) 0.25, (**b**) 0.75, (**c**) 1.00 e/f.u., (**d**) Li and (**e**) Na on monolayer 2H-MoS_2_. The red and green surfaces correspond to charge gains and loss of charge, respectively.

The obtained charge distributions of monolayer 2H-MoS_2_ injected with 0.25, 0.75, and 1.00 e/f.u. for the mono-layer MoS_2_ are shown in Fig. 4(a–c). The red and green surfaces correspond to gains and loss of charges, respectively. There is no apparent redistribution of charge for the MoS_2_ doped with electron injection concentrations of 0.25 e/f.u. or below. With increasing the electron injection concentrations, there is an apparent loss of electronic charges from the Mo-S bonds, whereas there is a net gain of electronic charge surrounding the Mo atoms. The distribution of electronic charge on the Mo atom shows an orbital characters of *d*z^2^ 59, indicating that the doped electrons and the lost electrons from the Mo-S bonds all fill the Mo *d*z^2^ orbital. The phenomenon of electron doping leading to occupation of the conduction band minimum (CBM) was also reported by Chakraborty *et al.*^60^. The transfer characteristic of the top-gated single-layer MoS_2_ transistor device showed an on-off ratio of ~10^5^ and a field-effect mobility of 50 cm^2^/Vs with electron doping of ~2 × 10^13^/cm^2^ 60. The differences of charge densities for the Li and Na adsorbed MoS_2_ systems are shown in Fig. 4(d,e), respectively. The electronic charge surrounding Li/Na decreases, resulting in a net loss of electronic charge of the Li/Na. There was a charge loss on the Mo-S bonds at the Li/Na adsorption site on MoS_2_. A net gain of electronic charge in the Li/Na-S bonds and Mo *d*z^2^ orbital can be observed. The Li/Na donate their electrons to the CBM of the 2H-MoS_2_^61^, which results in an *n*-type doping character of Li/Na adsorbed 2H-MoS_2_ systems. The same phenomenon has been reported Li-doped graphene systems^62^,^63^,^64^,^65^. The bonding of Li/Na adatoms appears to be primarily ionic bonding^66^, which is same with that in Li intercalated graphene system^67^,^68^.

**Figure 5:**
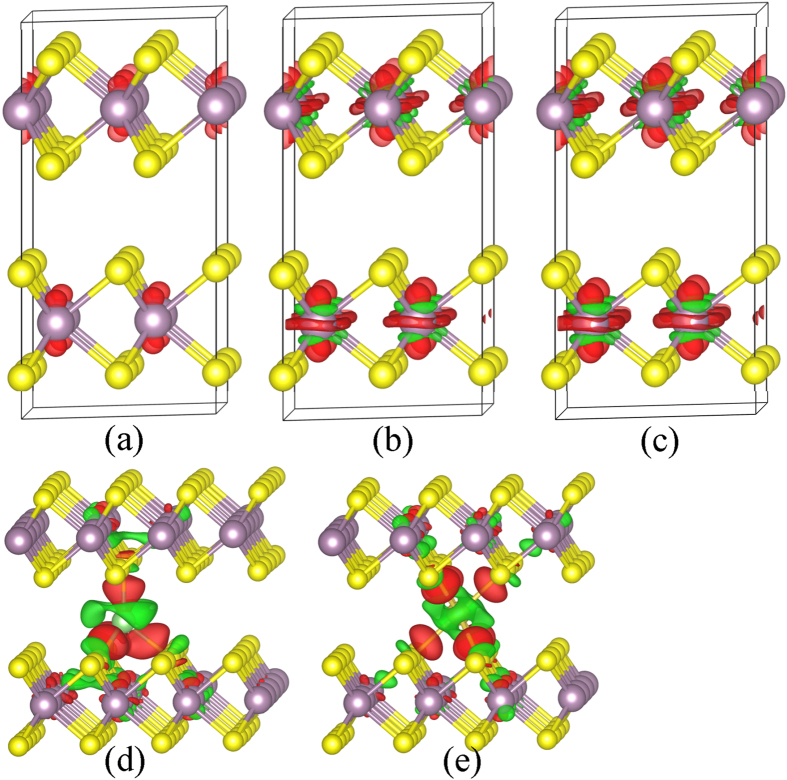
Charge distributions of bi-layer 2H-MoS_2_. Isosurface (0.003 e/Å^3^) of the charge distributions of 2H-MoS_2_ doped with (**a**) 0.25, (**b**) 0.75, (**c**) 1.00 e/f.u., (**d**) Li and (**e**) Na on bi-layers 2H-MoS_2_. The red and green surfaces correspond to charge gains and loss of charge, respectively.

The charge distributions of the bi-layers 2H-MoS_2_ injected with 0.25, 0.75, and 1.00 e/f.u. electron and Li/Na adsorption are shown in Fig. 5, which shows the same characteristics as those of the mono-layer 2H-MoS_2_.

## Discussion

**Figure 6:**
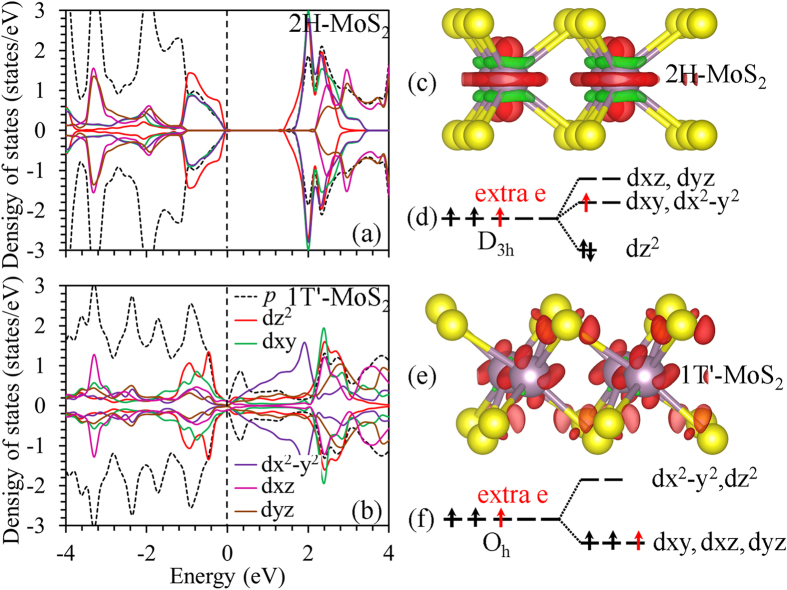
Orbital states. Partial density of states of (**a**) 2H- and (**b**) 1T’- monolayer MoS_2_. Isosurface (0.003 e/Å^3^) of the charge distributions of (**c**) 2H- and (**e**) 1T’- MoS_2_ doped with 1.00 e/f.u. Within crystal field theory, the Mo 4*d* orbitals (**d**) D_3h_- and (**f**) O_h_-MoS_6_ unit will split into three and two groups, respectively.

Within the framework of crystal field theory, the energy of the 4*d* orbitals of Mo ions will be affected by the arrangement of surrounding negative ions. The five 4*d* orbitals are initially degenerate (have the same energy). Placing six negatively charged ions at the vertices of an octahedron does not change the average energy of the 4*d* orbitals, but will remove their degeneracy. As the Mo atom is in trigonal prism coordination sites in the 2H-MoS_2_, the five degenerate 4*d* orbitals are split into (1) one singly degenerate state *dz*^2^ (filled), (2) two doubly-degenerate states *dx*^2^ − *y*^2^, *dxy* (empty), and (3) two doubly-degenerate states *dxz*, *dyz* (empty), as shown in Fig. 6(d). Whereas the Mo 4*d* orbitals of an O_h_-MoS_6_ unit in the 1T-MoS_2_ can be separated into two groups: (1) three degenerated *dxz*, *dyz* and *dxy* orbitals occupied by two electrons; and (2) non-occupied *dz*^2^ and *dx*^2^ − *y*^2^ as shown in Fig. 6(f). Incomplete occupation of the degenerated orbitals leads to the metallic ground state of the 1T-MoS_2_, and also decreases lattice stability compared with that of the 2H-MoS_2_^69^. As the 1T-MoS_2_ is doped with electrons, the extra electrons will occupy the *dxz*, *dyz* and *dxy* orbitals, thus increasing the stability of the 1T-MoS_2_. When such kind of doping occurs in the semiconducting 2H-MoS_2_, the extra electrons occupy the *dx*^2^ − *y*^2^ and *dxy* states, thus resulting in a metallic-like character of the electronic structure and destabilization of the lattice^31^.

The partial density of states (PDOS) of 2H- and 1T’- monolayer MoS_2_ are shown in Fig. 6(a,b), respectively. The 2H-monolayer MoS_2_ shows a semiconductor character with a band gap of 1.70 eV. The electronic states near the valence band maximum (VBM) and CBM are mainly composed of Mo 4*d*z^2^, 4*dx*^2^ − *y*^2^ and 4*dxy*, whereas the Mo 4*dxz* and 4*dyz* orbitals do not contribute to the energy states near the VBM and CBM, which agrees with the literature^17^,^18^,^70^. The 1T’- monolayer MoS_2_ shows a metallic-like character. The extra electrons either from injection or from ion intercalation doping occupy the Mo 4*d*z^2^, and induce loss of charges from the Mo-S bonds, which will destabilize the lattice of the 2H-MoS_2_ as shown in Fig. 6(c). On the contrary, there is no loss of charge from the Mo-S bonds in the 1T’-MoS_2_.

From the charge distribution shown in Fig. 6(e), the extra electrons occupy the S 3*p* and Mo orbitals of *dxz*, *dyz* and *dxy*^59^. This explains the stabilization of the 1T’ structure upon Li/Na adsorption or electron doping. The electron doping destabilizes the crystal structure of the 2H-MoS_2_, and causes the structural transformation into the 1T’ phase through the re-distribution of the Mo 4*d* orbitals.

## Conclusion

The stability of 2H- and 1T’-MoS_2_ for both the mono- and bi-layers upon electron doping was investigated using the density functional theory, and then linked with that for Li/Na intercalation process. After doping with electrons, the 2H- and 1T’-MoS_2_ show semiconductor and metallic characters, respectively. The extra electrons either from charge injection or from ion intercalation doping occupy the Mo 4*dz*^2^ in 2H-MoS_2_, and induce loss of electronic charge from the Mo-S bonds. Whereas, the extra electrons occupy the S 3*p* and Mo orbitals of *dxz*, *dyz* and *dxy* in the 1T’-MoS_2_ without apparent loss of electronic charge from the Mo-S bonds. Whereas electron doping destabilizes the crystal structure of the 2H-MoS_2_, and causes its structural transformation into the 1T’ phase through the redistribution of the Mo 4*d* orbitals. The critical values of electron concentrations for the 2H→1T’ phase transition decrease with increasing the layer thickness.

### Simulation details

The stability of 2H- and 1T’-MoS_2_ and Li/Na adsorption behavior in the two polytypic structures were investigated using first principles plane-wave simulations based on DFT as implemented in the Vienna *ab initio* simulation package (VASP)^71^. Electron-ion interaction and electron exchange-correlation were described using the projector augmented wave (PAW) method^72^ and the generalized gradient approximation was described using the Perdew-Burke-Ernzerhof (PBE) function, respectively. An energy cutoff of 520 eV was used for the plane wave basis sets. Spin-polarization was considered applied for all the simulations.

A 2 × 2 supercell of MoS_2_ monolayer was used to investigate the stability of 2H- and 1T’ phases with mono- and bi-layers of MoS_2_. A 6 × 6 supercell of MoS_2_ monolayer was used to investigate the adsorption of Li/Na. A 25 Å vacuum space were constructed to avoid the periodical image interactions between two adjacent MoS_2_ layers. The Brillouin zone was integrated using the Monkhorst-Pack scheme^73^ with 5 × 5 × 1 *k*-grid. All the atomic positions and cell parameters were relaxed until the force on each atom is less than 0.02 eV/Å. Electron concentrations of 0.125–1.00 e/f.u., i.e. 0.14–1.13 × 10^15^/cm^2^ and 0.28–2.26 × 10^15^/cm^2^ were injected into the mono- and bi-layer MoS_2_, respectively, to investigate the stability of 2H- and 1T’-MoS_2_.

## Additional Information

**How to cite this article**: Sun, X. *et al.* Origin of Structural Transformation in Mono- and Bi-Layered Molybdenum Disulfide. *Sci. Rep.* **6**, 26666; doi: 10.1038/srep26666 (2016).

## Figures and Tables

**Table 1:** Calculated adsorption energies (in eV) versus vacuum (V) and bulk metal (B) reference states for Li and Na in mono- and bi-layers MoS_2._

	H		T		Octahedral		Tetrahedral	
	V	B	V	B	V	B	V	B
monolayer								
Li	−1.64	−0.03	−1.79	−0.18				
Na	−1.19	−0.10	−1.27	−0.18				
bilayer								
Li	−1.80	−0.19	−2.01	−0.40	−2.49	−0.88	−2.74	−1.13
Na	−1.56	−0.47	−1.54	−0.45	−1.65	−0.56	−1.53	−0.44
